# The effect of tetrandrine combined with cisplatin on proliferation and apoptosis of A549/DDP cells and A549 cells

**DOI:** 10.1186/s12935-017-0410-1

**Published:** 2017-03-27

**Authors:** Ling-Yun Ye, Song Hu, Hua-E Xu, Rong-Rong Xu, Hui Kong, Xiao-Ning Zeng, Wei-Ping Xie, Hong Wang

**Affiliations:** 1 Department of Respiratory Medicine, The First Affiliated Hospital of Nanjing Medical University, 300 Guangzhou Road, Nanjing, 210029 Jiangsu People’s Republic of China; 2 Department of Pharmacy, The First Affiliated Hospital of Nanjing Medical University, 300 Guangzhou Road, Nanjing, 210029 Jiangsu People’s Republic of China

**Keywords:** Tetrandrine, Non-small cell lung cancer, Proliferation, Apoptosis, Autophagy

## Abstract

**Background:**

Non-small cell lung cancer comprises the majority of lung cancer cases and is insensitive to chemotherapy. Most patients develop drug resistance. Recently, tetrandrine (TET), a bis-benzylisoquinoline alkaloid, was identified as a novel anti-cancer agent. However, the effect of tetrandrine combined with cisplatin on lung cancer has not yet been studied. We aimed to identify a possible synergistic effect between tetrandrine and cisplatin, besides, to investigate the effects of TET in combination with DDP on proliferation and apoptosis in cisplatin-resistant and cisplatin-sensitive A549 cell lines, and to study the underlying mechanism.

**Methods:**

Cell viability was confirmed with CCK8 assays, and the IC_50_ values for each treatment group were calculated. The synergistic interaction of these drugs was evaluated using an isobolographic analysis. Proliferation was assessed by EDU staining. Hoechst staining and flow cytometry were used to assess apoptosis. Apoptosis- and autophagy-associated proteins were analyzed by western blot. Transmission electron microscopy was used to detect autophagy, RFP-GFP-LC3 lentivirus was used to perform autophagic flux assay.

**Results:**

Tetrandrine and cisplatin exerted synergistic cytotoxic effects on both cisplatin-resistant and cisplatin-sensitive A549 cell lines. The combination of tetrandrine and cisplatin induced apoptosis and inhibited proliferation in a synergistic manner. The formation of autophagosomes was evident by transmission electron microscopy. The autophagic flux of combination treatment was increased.

**Conclusions:**

Tetrandrine synergized with cisplatin to reduce the viability of cisplatin-resistant and cisplatin-sensitive A549 cells, tetrandrine could reverse the resistance of A549 cells to cisplatin. Tetrandrine combined with cisplatin could induce autophagy. Therefore, tetrandrine is a potent autophagy agonist and may be a promising drug for the treatment of non-small cell lung cancer.

## Background

Lung cancer is one of the most common malignant tumors and remains the leading cause of cancer-related death worldwide. Non-small cell lung cancer (NSCLC) comprises the majority of lung cancer cases [1]. The most powerful prognosticator of clinical outcome for patients with NSCLC is the response to neoadjuvant chemotherapy. Although this is the best available at present, it remains impossible to predict which patients will respond to chemotherapy. Moreover, most NSCLC tumors develop drug resistance [2]. Because cisplatin-based chemotherapy has been the first-line treatment for NSCLC in the clinic [3], the effect of cisplatin on lung cancer has been widely studied. It has been shown that the anticancer effect of cisplatin depends on its ability to generate irreparable DNA lesions. However, the clinical responses elicited by cisplatin in lung cancer patients usually vanish because of resistance to the cytotoxic activity of cisplatin [4–7]. Therefore, new drugs are required to reverse acquired drug resistance to improve survival and quality of life. Previous studies have shown that several plant polyphenols can achieve this goal [8, 9].

In recent years, tetrandrine (TET) has garnered increasing attention due to its potential use as a novel anticancer agent either alone or in combination with other chemotherapeutic drugs [10–16]. TET, a bis-benzylisoquinoline alkaloid that is extracted from the root tuber of the Chinese herb *Stephania tetrandra* S. Moore, has been widely used to treat arthritis, arrhythmia, inflammation, silicosis and various types of cancer [17–19]. Studies have demonstrated that TET can cause cell-cycle arrest and induce apoptosis in A549 human lung carcinoma cells [20]. Moreover, the inhibition of extracellular signal-regulated kinase (ERK) synergistically enhanced TET-induced apoptosis in A549 cells [21]. However, whether TET enhances the efficacy of chemotherapy against lung cancer remains to be elucidated. In this study, we investigated the synergistic effects between TET and cisplatin.

Apoptosis and autophagy control basic cellular metabolism. All tumor cells undergo apoptosis, which is also known as programmed cell death, especially in the vicinity of tumor necrosis. Most chemotherapy drugs rely on the induction of apoptosis for efficacy [22]. Autophagy is a cellular pathway involved in protein and organelle degradation in the lysosome that serves as a type of cellular renovation. Three types of autophagy have been identified: macro-autophagy, micro-autophagy and chaperone-mediated autophagy [23]. Autophagic dysfunction is associated with several pathological processes, such as cancer, microbial infection, aging and neurodegeneration [24]. Several synthetic chemotherapeutic agents induce autophagic cell death in a variety of cancer cells. Autophagy was recently shown to block the induction of apoptosis, and apoptosis-associated activation was shown to deactivate the autophagic process. Paradoxically, autophagy may also help induce apoptosis in some cases [25]. Therefore, autophagy and the relationship between apoptosis and autophagy need to be further explored to manipulate these pathways for the treatment of human disease.

Thus, we aimed to identify possible synergism between TET and cisplatin; furthermore, we sought to investigate the effects of TET in combination with DDP on proliferation and apoptosis in cisplatin-resistant and cisplatin-sensitive A549 cell lines, and to find the role of autophagy in the drug treatments.

## Results

### TET and cisplatin display independent and synergistic cytotoxicity against cisplatin-resistant and cisplatin-sensitive A549 cells

**Fig. 1:**
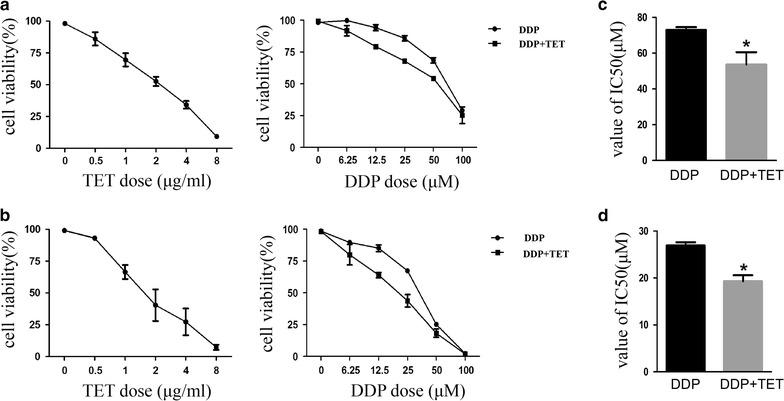
Dose-response curves of DDP, TET and the combination treatment. Cisplatin-resistant (**a**) and cisplatin-sensitive (**b**) A549 cells were treated with different concentrations of the two drugs (TET: 0.5–8 µg/ml; DDP: 6.25–100 µM) for 48 h. The concentration of TET in the combination treatment was 0.25 µg/ml. C and D are the IC_50_ values of DDP and combination treatment. *p < 0.05 versus the DDP treatment (n = 3)

**Fig. 2:**
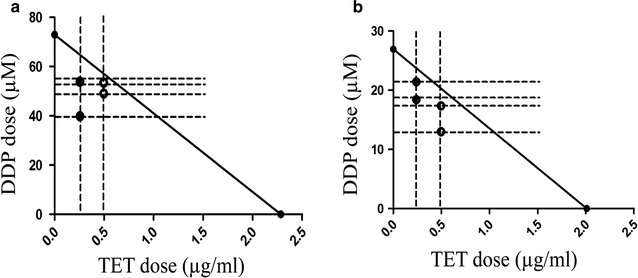
Isobolographic analysis of the cytotoxic effect of the combination treatment on the two cell lines. **a** The synergistic effect of the combination treatment on cisplatin-resistant A549 cells. **b** The synergistic effect of the combination treatment on cisplatin-sensitive A549 cells. The *solid* and *hollow dots* represent the IC_50_ values of the combination treatments at 0.25 and 0.5 µg/ml TET, respectively

To determine the effect of TET and DDP on cell viability, cells treated with different concentrations of the two drugs (TET: 0.5–8 µg/ml; DDP: 6.25–100 µM) for 48 h were subjected to a CCK8 assay. The IC_50_ of each agent was calculated using dose–response curves (Fig. 1). Based on the IC_50_ of TET, two low doses (0.25 and 0.5 µg/ml) were selected for combination treatments with DDP. Isobolograms were generated using the IC_50_ values (Fig. 2). Both DDP and TET dose-dependently inhibited viability. Compared with DDP treatment alone, the combination treatment significantly reduced cell viability, and the isobolograms illustrated the significant synergistic effect of the combination treatment. These data suggest that TET could partially reverse cisplatin resistance.

### TET increased the cisplatin-induced inhibition of proliferation in cisplatin-resistant and cisplatin-sensitive A549 cells

**Fig. 3:**
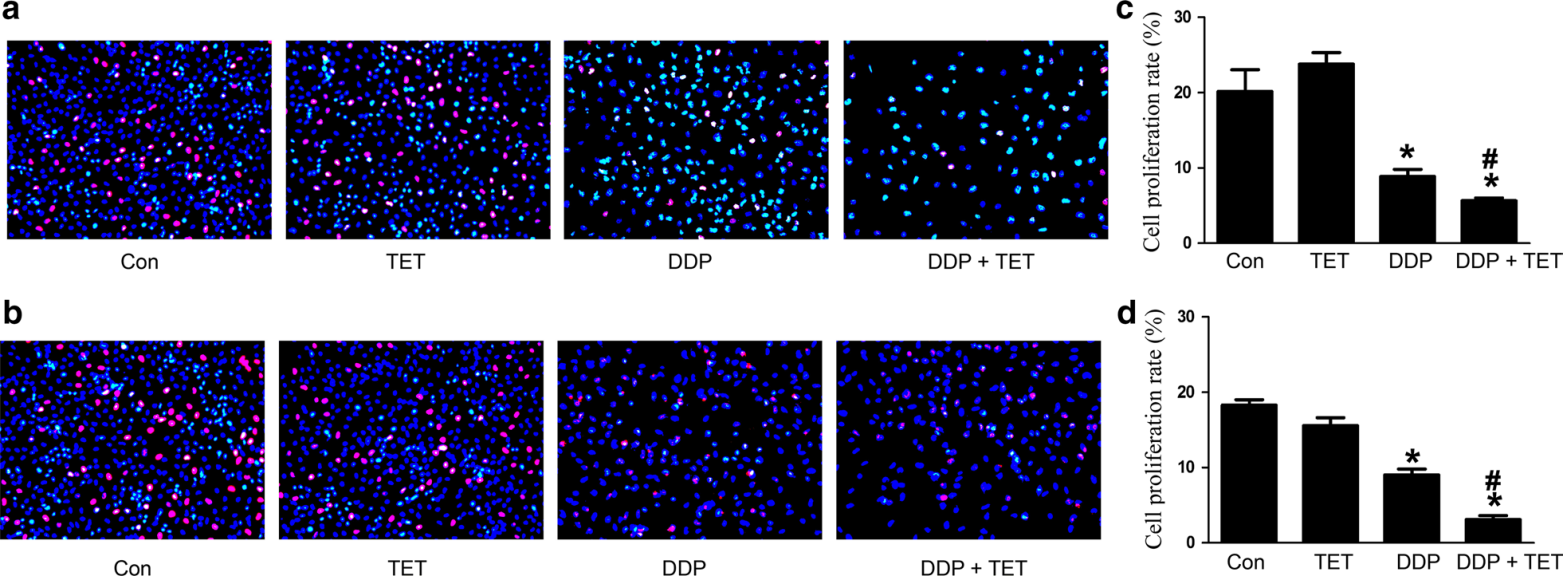
Influence of each treatment on cell proliferation. **a** EDU staining of cisplatin-resistant A549 cells after various treatments (×200). The concentrations of DDP and TET were 60 µM and 0.25 µg/ml, respectively. **b** EDU staining of cisplatin-sensitive A549 cells (×200). The concentrations of DDP and TET were 25 µM and 0.25 µg/ml, respectively. The time of the drug treatments was 12 h. **c**, **d** are the statistical analyses of **a**, **b**. *p < 0.05 versus the control cells; ^#^p < 0.05 versus DDP treatment (n = 3)

To determine the effect of TET and DDP on proliferation, we exposed cisplatin-resistant and cisplatin-sensitive A549 cells to TET alone, DDP alone or their combination for 12 h. EDU staining was performed to detect proliferating cells. As shown in Fig. 3, compared with the control group and the DDP group, cell proliferation decreased significantly when TET was combined with DDP (p < 0.05). Furthermore, the cell lysates were analyzed by western blot to evaluate the expression of proliferation-related proteins (Fig. 6). The combination treatment significantly decreased p-Akt expression.

### TET enhanced cisplatin-mediated apoptosis in cisplatin-resistant and cisplatin-sensitive A549 cells

**Fig. 4:**
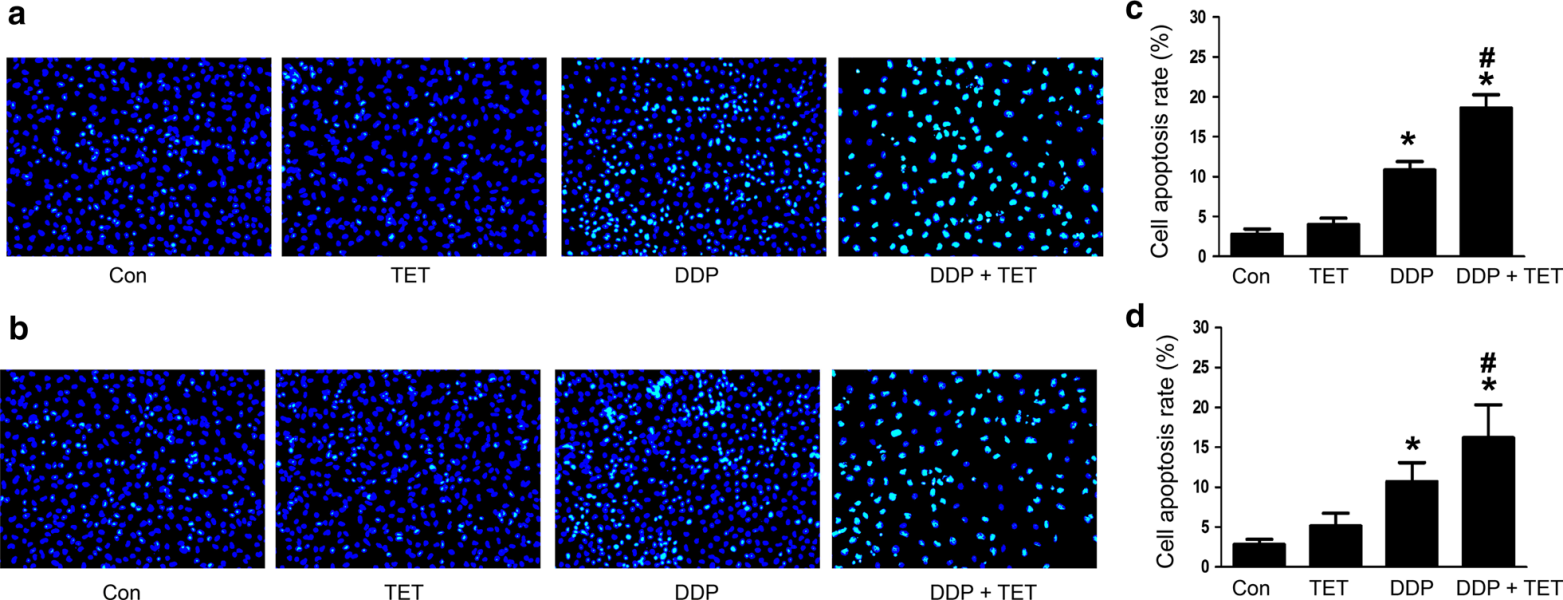
Influence of each treatment on apoptosis. **a** Hoechst staining of cisplatin-resistant A549 cells after various treatments (×200). The concentrations of DDP and TET were 60 µM and 0.25 µg/ml, respectively. **b** Hoechst staining of cisplatin-sensitive A549 cells (×200). The concentrations of DDP and TET were 25 µM and 0.25 µg/ml, respectively. The time of the drug treatments was 12 h. **c**, **d** are the statistical analyses of **a**, **b**. *p < 0.05 versus the control cells; ^#^p < 0.05 versus DDP treatment (n = 3)

**Fig. 5:**
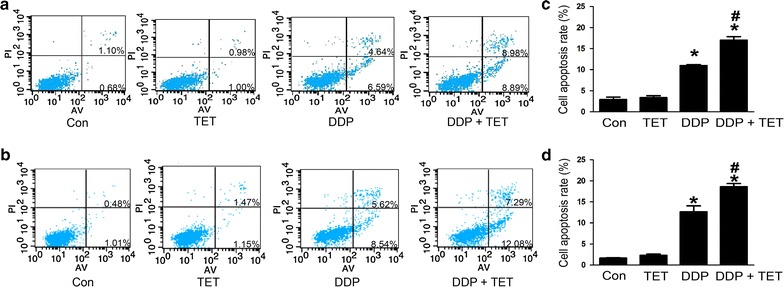
Apoptosis induced by each treatment for 12 h on cisplatin-resistant A549 cells (**a**) and cisplatin-sensitive A549 cells (**b**). The apoptotic cells include both early apoptotic and late apoptotic cells. **a** The concentrations of DDP and TET were 60 µM and 0.25 µg/ml, respectively. **b** The concentrations of DDP and TET were 25 µM and 0.25 µg/ml, respectively. **c**, **d** are the statistical analyses of **a**, **b**. *p < 0.05 versus the control cells, ^#^p < 0.05 versus the DDP treatment (n = 3)

**Fig. 6:**
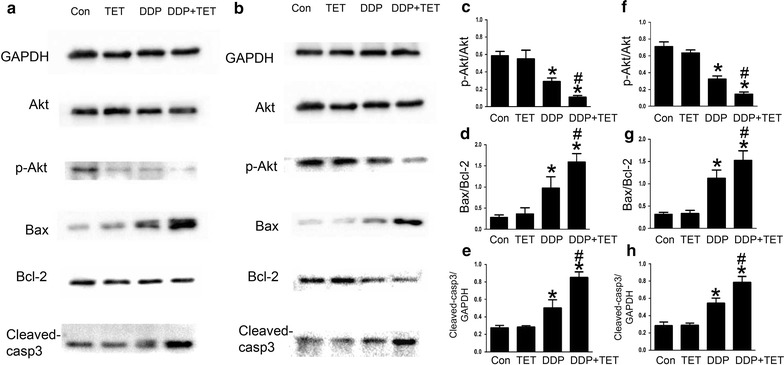
The expression of proliferation- and apoptosis-related proteins of the cells in response to different treatments for 12 h. **a** Proteins expression of cisplatin-resistant A549 cells after each drug treatment. The concentrations of DDP and TET were 60 µM and 0.25 µg/ml, respectively. **b** Proteins expression of cisplatin- sensitive A549 cells. The concentrations of DDP and TET were 25 µM and 0.25 µg/ml, respectively. **c**–**e** are the statistical analyses of **a**, besides, **f**–**h** are the statistical analyses of **b**. *p < 0.05 versus the control cells, ^#^p < 0.05 versus the DDP treatment (n = 3)

To further evaluate the effect of TET on DDP-mediated apoptosis, cisplatin-resistant and cisplatin-sensitive A549 cells were treated as described above. Hoechst staining and annexin V/propidium iodide (PI) staining were performed to observe apoptosis. Western blot analysis was used to investigate the levels of intracellular apoptosis-related proteins. Figures 4 and 5 show that the apoptosis rate in response to treatment with DDP alone or the combination was significantly higher than that in response to treatment with control or TET alone (p < 0.05). Moreover, the combination treatment increased apoptosis compared with DDP treatment alone (p < 0.05). In the combination treatment group, Bax and cleaved-caspase 3 were upregulated, whereas Bcl-2 was downregulated (Fig. 6).

### Autophagy and autophagic flux detection in each treatment group

**Fig. 7:**
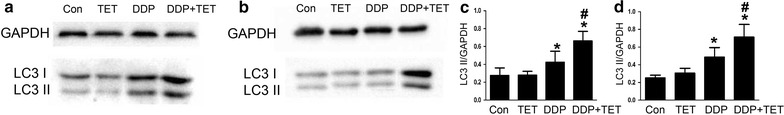
LC3 II protein expression levels of cisplatin-resistant A549 cells (**a**) and cisplatin-sensitive A549 cells (**b**). The concentrations of DDP in **a**, **b** were 60 and 25 µM, respectively. The concentrations of TET was 0.25 µg/ml. The time of the drug treatments was 12 h. **c**, **d** are the statistical analyses of **a**, **e** and **f** are the statistical analyses of **b**. *p < 0.05 versus the control cells, ^#^represents p < 0.05 versus the DDP treatment (n = 3)

**Fig. 8:**
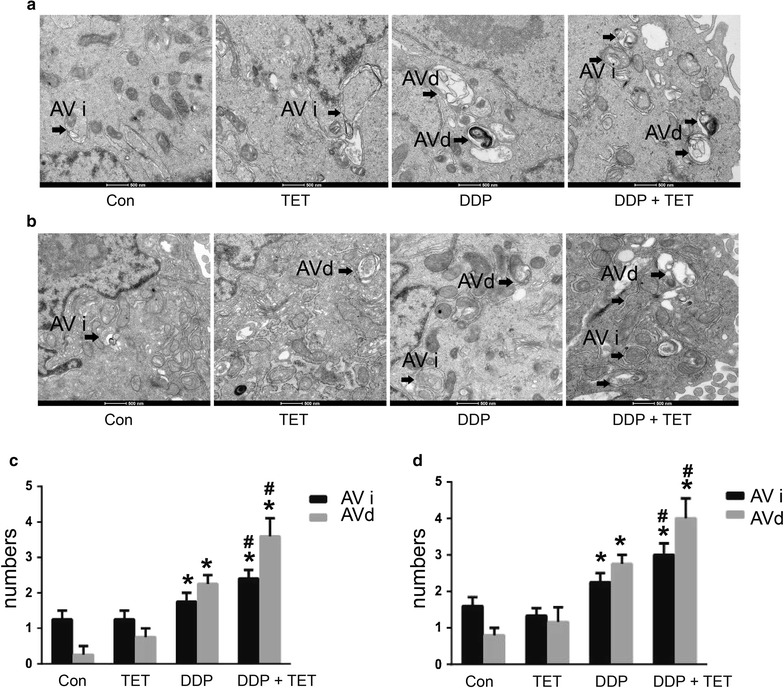
TEM images of autophagosomes. Autophagy induced by each treatment in cisplatin-resistant A549 cells (**a**) and cisplatin-sensitive A549 cells (**b**). The concentrations of DDP in **a**, **b** were 60 and 25 µM, respectively. The concentration of TET was 0.25 µg/ml. The time of the drug treatments was 12 h. The *scale bars* represent 500 nm, and the *arrows* indicate the autophagosomes. **c**, **d** are the statistical analyses of **a**, **b**. *p < 0.05 versus the control cells, ^#^p < 0.05 versus the DDP treatment (n = 3)

**Fig. 9:**
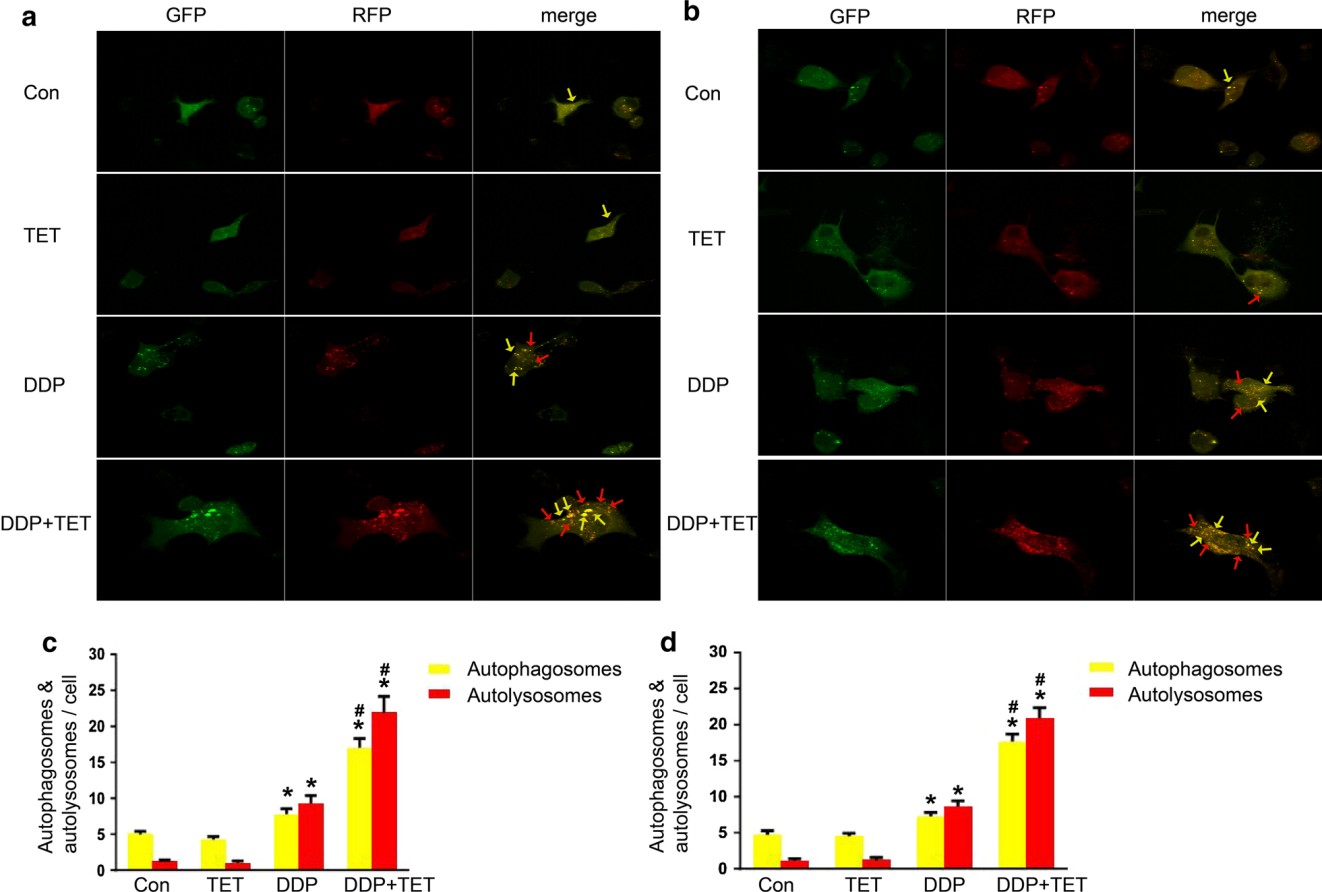
Autophagic flux assay of cisplatin-resistant A549 cells (**a**) and cisplatin-sensitive A549 cells (**b**) (×400). The concentrations of DDP in **a**, **b** were 60 and 25 µM, respectively. The concentration of TET was 0.25 µg/ml. The time of the drug treatments was 12 h. *Yellow arrows* point to autophagosomes and *red arrows* point to autolysosomes. **c**, **d** are the statistical analyses of **a**, **b**. *p < 0.05 versus the control cells, ^#^p < 0.05 versus the DDP treatment (n = 3)

Western blot analysis was used to determine the expression of the autophagy marker protein LC3. Figure 7 shows that the production of LC3 significantly increased in combination treatment group. To further confirm the occurrence of autophagy, the formation of autophagosomes was directly observed using transmission electron microscopy (TEM) (Fig. 8). Besides, Fig. 9 shows that the autophagic flux was increased in combination treatment. Taken together, these observations indicated that TET could significantly increase autophagy when combined with DDP.

## Discussion

Our research demonstrated that TET dose-dependently inhibited the viability of cisplatin-resistant and cisplatin-sensitive A549 cells and increased the sensitivity of cisplatin-resistant A549 cells to cisplatin. Previous studies have shown that TET can induce apoptosis by activating caspase-3 in lung carcinoma cells [20]. Our study showed that DDP treatment alone downregulated the expression of Bcl2 and upregulated the expression of Bax, indicating that DDP can induce caspase-dependent apoptosis, when combined with TET, this effect is more obvious. Compared with DDP treatment alone, the combination of TET and DDP significantly decreased A549 cell proliferation and increased apoptosis. These data suggest that TET synergizes with DDP in both cell lines and that the anti-cancer effect depends on the inhibition of proliferation and the promotion of apoptosis.

In addition, the combination treatment downregulated the expression of p-Akt, suggesting that the synergistic effect was accompanied by the inhibition of the PI3K/Akt signaling pathway. The PI3K/Akt pathway is necessary for the regulation of various biological processes, such as survival, proliferation, apoptosis and differentiation [26]. Thus, many anticancer therapeutic strategies have focused on blocking this pathway. Earlier studies have shown that PI3K/Akt signaling pathway activation is closely related to drug resistance [27, 28]. TET can repress Akt activity in cancer cells; thus, TET, a type of PI3K/Akt inhibitor, may be used to develop new anti-cancer treatment strategies.

Apoptosis serves as a natural barrier to the development of cancer [29]. Previous studies have shown that the resistance of NSCLC cells to various cytotoxic therapies may be due to a defect in apoptosis signaling [30]. Bcl-2 family proteins play a pivotal role in the regulation of apoptosis. Some reports have indicated that the absence of Bax blocks apoptosis and increases drug resistance [31]. We found that the expression of the pro-apoptotic protein Bax was upregulated and that the expression of the anti-apoptotic protein Bcl-2 was decreased in DDP-treated cells. The combination of TET and DDP enhanced this effect, suggesting that TET could enhance cisplatin-mediated apoptosis via the Bax/Bcl-2 pathway, which reversed the resistance of cisplatin-resistant A549 cells. The relationship between Bax/Bcl-2 and drug resistance needs to be further investigated.

Because apoptosis is the most common target of chemotherapy, the significance of autophagy in antitumor management has not received considerable attention [32]. However, over the past decade, interest in the roles of autophagy in human health and disease has become widespread [33]. Autophagy, meaning “self-eating,” is a cellular degradation pathway in which cytoplasmic cargos are delivered to the lysosome, and intracellular components are recycled. Autophagy occurs at low levels in almost all cells, but it is rapidly upregulated when cells need to generate nutrients and energy, such as during starvation, high bioenergetic demand or growth factor withdrawal [34]. To some extent, it represents cellular adaptation to stress and serves as a protective mechanism. However, autophagy is a double-edged sword, and it is now widely implicated in pathophysiological processes, such as cancer. Autophagy may influence the initiation and progression of cancer as well as therapeutic interventions [35]. Previous studies have indicated that autophagy results in the excessive degradation of the cytoplasm, leading to a form of non-apoptotic programmed cell death called ‘autophagic’ cell death [36]. As described above, our research showed that the expression of the autophagy marker protein LC3-II was upregulated and the autophagic flux was increased by the combination treatment, autophagosome formation was observed by TEM. These results suggest that drug treatment could induce autophagy and that autophagy may participate in the resistance of cells to cisplatin. These details need to be elucidated in future studies, for example, autophagy inhibitor or genetic approach to knockdown or knockout core Atgs could be used to verify the role of autophagy in the drug treatments.

Recent years, more and more researches study the relationship between autophagy and apoptosis. Autophagy and apoptosis may occur concurrently in a cell, and autophagy can facilitate the activation of apoptosis in some cases, though it suppresses apoptosis in most instances [36]. Individual genes, such as ATG genes, may play an essential role in the pro-apoptotic signaling pathway [37]. Autophagy may stimulate apoptosis via the formation of autophagosomes, and this process plays an important role in the activation of caspase-8 [38]. Moreover, autophagy can deplete endogenous inhibitors of apoptosis to trigger this cell death pathway [36]. LC3 is a major regulator of autophagosome formation; we found that the combination treatment upregulated LC3-II, which indicated that autophagy may stimulate apoptosis via the formation of autophagosomes. Some studies have shown that the Bcl-2 family of proteins also plays a vital role in autophagy regulation and that Bcl-2 can inhibit autophagy [39]. Our research showed that the combination treatment downregulated Bcl-2 expression, thus supporting the role of Bcl-2 family proteins in autophagy. The relationship between these two types of cell death is complex, and autophagy-apoptosis crosstalk has a broad pathophysiological influence. Therefore, an improved understanding of these pathways may help to yield additional therapeutic targets for lung cancer.

Other studies have demonstrated that TET induces the accumulation of reactive oxygen species (ROS) [40], and the generation of ROS plays an important role in the regulation of apoptosis and autophagy [41, 42]. The results described herein may only represent part of the effect of TET. Further studies are essential to better understand these molecular mechanisms and to obtain new targets for cancer treatment.

## Conclusions

In our study, we discovered that TET increased the sensitivity of cisplatin-resistant A549 cells to cisplatin, and the combination of TET and DDP inhibited cell proliferation while increasing apoptosis. Additionally, the combination treatment could induce autophagy. All of these findings may provide a new perspective on the treatment of lung cancer.

## Methods

### Cell culture

The cells were purchased from the Institute of Biochemistry and Cell Biology of the Chinese Academy of Sciences (Shanghai, China). The cells were propagated in RPMI 1640 medium (GIBCO BRL) supplemented with 10% fetal bovine serum, 100 U/ml penicillin and 100 U/ml streptomycin at 37 °C in a 5% CO_2_ humidified atmosphere. To maintain the resistance of cisplatin-resistant A549 cells, 2 µg/ml cisplatin was added to the medium for these cells. The cells were cultured in complete medium without cisplatin for 3 days before any experiment was performed.

### Reagents

TET was kindly provided by Zhejiang Haizheng Pharmaceutical Co., Ltd. Cisplatin was obtained from the First Affiliated Hospital of Nanjing Medical University. The EDU and Hoechst staining solutions were purchased from Guangzhou RiboBio Co. (Guangzhou, China). The annexin V-fluorescein isothiocyanate (FITC) kit was purchased from Bender MedSystems (Vienna, Austria). The RFP-GFP-LC3 lentivirus was purchased from Shanghai Genechem Co., Ltd.

### Cell viability assay

Cell viability was measured using a CCK8 assay kit (Obio Technology (Shanghai) Co., Ltd.). The cells (1 × 10^3^ cells/well) were seeded in 96-well plates, grown overnight, and then treated with the indicated concentrations of different drugs for 48 h. Subsequently, 10 µl of CCK8 solution was added to each well, and the plates were incubated for 1 h. The absorbance was measured at 450 nm using a microplate reader (BioTek, Winooski, VT, USA).

### Isobolographic analysis

Synergism between TET and DDP was evaluated with an isobolographic analysis. Briefly, the doses of TET were plotted on the x-axis, and the doses of DDP were plotted on the y-axis; points representing equal effects on cell viability were connected to obtain an isobologram. The 50% inhibitory concentration (IC_50_) of TET plotted on the x-axis was connected to that of DDP plotted on the y-axis to obtain a straight line. Points on, below or above the line indicated an additive, synergistic or antagonistic effect, respectively.

### EDU staining for cell proliferation

The cells were treated with different drugs, washed three times with PBS, and then stained with 300 µl of staining solution for 2 h according to the manufacturer’s protocol. After an additional three washes with PBS, the cells were examined with a fluorescence microscope (Olympus, Japan). Proliferating cells were identified by red staining, and the number of proliferating cells in ten different fields was counted.

### Hoechst 33342 staining for apoptosis analysis

The cells were seeded on microscope slides (Millipore, USA) and treated with different drugs for 12 h. After three washes with PBS, the cells were incubated with Hoechst staining buffer for 10 min at room temperature in the dark. The percentage of cells undergoing apoptosis was then determined with a fluorescence microscope (Olympus, Japan). Apoptotic cells were identified by bright blue staining, and the number of apoptotic cells in ten different fields was counted.

### Apoptosis assay by flow cytometry

The cells were harvested after drug treatment, washed with PBS, and then stained with annexin V-FITC and PI according to the manufacturer’s protocol. After incubation in the dark for 15 min, the cells were analyzed using a FACScan flow cytometer (Becton–Dickinson, CA, USA).

### Western blot analysis

The cells were harvested and lysed in RIPA lysis buffer (Thermo Scientific, Rockford, IL, USA) supplemented with a protease inhibitor cocktail (Roche, Indianapolis, IN, USA). Protein from each sample was resolved by 10% sodium dodecyl sulfate-polyacrylamide gel electrophoresis (SDS-PAGE) and transferred to polyvinylidene fluoride (PVDF) membranes (Millipore, Billerica, MA, USA). The membranes were blocked with 5% nonfat milk (Bio-Rad) in Tris-buffered saline containing 0.1% Tween 20 (TBST) at room temperature for 1 h and then incubated with anti-pAKT (1:1000, Cell Signaling Technology, Inc., Beverly, MA, USA), anti-AKT (1:1000, Cell Signaling Technology), anti-Bax (1:1000, Cell Signaling Technology), anti-Cleaved caspase 3 (1:1000, Cell Signaling Technology), anti-Bcl-2 (1:1000, Abcam, Cambridge, UK), anti-glyceraldehyde-3 phosphate dehydrogenase (GAPDH) (1:5000; Bioworld, Nanjing, China) primary antibody at 4 **°**C overnight. After washing with TBST, the membranes were incubated with HRP-conjugated secondary antibody for 1 h at room temperature. Following three additional washes with TBST, the protein bands of interest were visualized using enhanced chemiluminescence detection reagents (Thermo) and the Bio-Rad Gel Doc/ChemiDoc Imaging System and then analyzed using Quantity One software.

### Transmission electron microscopy analysis and autophagic flux assay

Standard TEM was conducted to analyze cellular ultrastructure. Approximately 12 h after drug treatment, the cells were fixed and embedded. Thin sections (90 nm) were examined at 80 kV with a JEOL 1200EX transmission electron microscope. Approximately 15 cells were counted, and autophagosomes were defined as double-membrane vacuoles measuring 0.5 or 200 µm. The cells were seeded on the culture plate, and moderate RFP-GFP-LC3 lentivirus were added to the plate according to the instructions, the cells were propagated in RPMI 1640 medium supplemented with 10% fetal bovine serum, 100 U/ml penicillin and 100 U/ml streptomycin at 37 °C in a 5% CO_2_ humidified atmosphere for 72 h. Then the cells transfected with RFP-GFP-LC3 lentivirus were treated with different drugs for 12 h, and analysed by laser confocal microscope (ZEISS LSM, German).

### Statistical analysis

All of the experiments were performed at least three times. Data are presented as the mean ± SD. All of the statistical analyses were performed using one-way analysis of variance (ANOVA) followed by Dunnett’s post hoc test employing Prism 6.00 software (GraphPad Software, San Diego, CA, USA) and SPSS version 20 (SPSS Inc., Chicago, IL, USA). P < 0.05 was considered to indicate a significant difference.

## Abbreviations

NSCLC: non-small cell lung cancer
DDP: *cis*-diammineplatinum dichloride
TET: tetrandrine
EdU: 5-ethynyl-2′-deoxyuridine
FITC: fluorescein isothiocyanate
CCK8: cell counting kit-8
PI: propidium iodide
SDS-PAGE: sodium dodecyl sulfate-polyacrylamide gel electrophoresis
PVDF: polyvinylidene fluoride
TBST: tris-buffered saline containing 0.1% Tween 20
GAPDH: glyceraldehyde-3 phosphate dehydrogenase
LC3: light chain 3
TEM: transmission electron microscopy
ANOVA: analysis of variance
ROS: reactive oxygen species
